# Case Report: Surgical Therapy for Left Innominate Vein Aneurysm Under Thoracoscopy

**DOI:** 10.3389/fsurg.2021.741840

**Published:** 2021-11-12

**Authors:** Yan Hu, Siying Ren, Chao Zeng, Jina Li, Min Zou, Li Wang, Peng Xiao, Fenglei Yu, Wenliang Liu

**Affiliations:** ^1^Department of Thoracic Surgery, The Second Xiangya Hospital of Central South University, Changsha, China; ^2^Department of Respiratory and Critical Care Medicine, The Second Xiangya Hospital of Central South University, Changsha, China; ^3^Department of Cardiothoracic Surgery, The Third Xiangya Hospital of Central South University, Changsha, China

**Keywords:** left innominate vein aneurysm, surgery, thoracoscopy, Castleman's disease, thymoma

## Abstract

Left innominate vein aneurysm is extremely rare, with a limited number of case reports present in the literature. Herein, we report a case of a 50-year-old female patient presenting with an incidental finding of an anterior mediastinal mass on chest radiography during a routine health examination. Contrast-enhanced computerized tomography (CT) of the chest showed a 4.8 × 4.6 cm anterior mediastinal mass with significant homogenous enhancement after injection of the contrast medium, suggesting a diagnosis of Castleman's disease, but not excluding thymoma. The patient underwent surgical resection of the anterior mediastinal mass under a thoracoscopic approach. Postoperative pathology confirmed the diagnosis of a left innominate vein aneurysm. This is the first case reporting a left innominate vein aneurysm resected under thoracoscopy. Despite this successful treatment experience, we need to emphasize that open thoracotomy or median sternotomy should be chosen as the first choice for surgeons who lack experience in thoracoscopic surgery, with the aim of avoiding intraoperative accidents.

## Introduction

Mediastinal venous aneurysm is extremely rare and can be easily misdiagnosed (1). Its etiology is unknown and it often presents no obvious clinical symptoms (2). This type of anomaly may originate from the superior vena cava, innominate vein, or azygos vein. There are a few case reports on the surgical treatment of left innominate vein aneurysm; however, none of these cases were surgically resected using the thoracoscopic approach. Herein, we report a case of a left innominate vein aneurysm resected under thoracoscopy.

## Case Presentation

**Figure 1:**
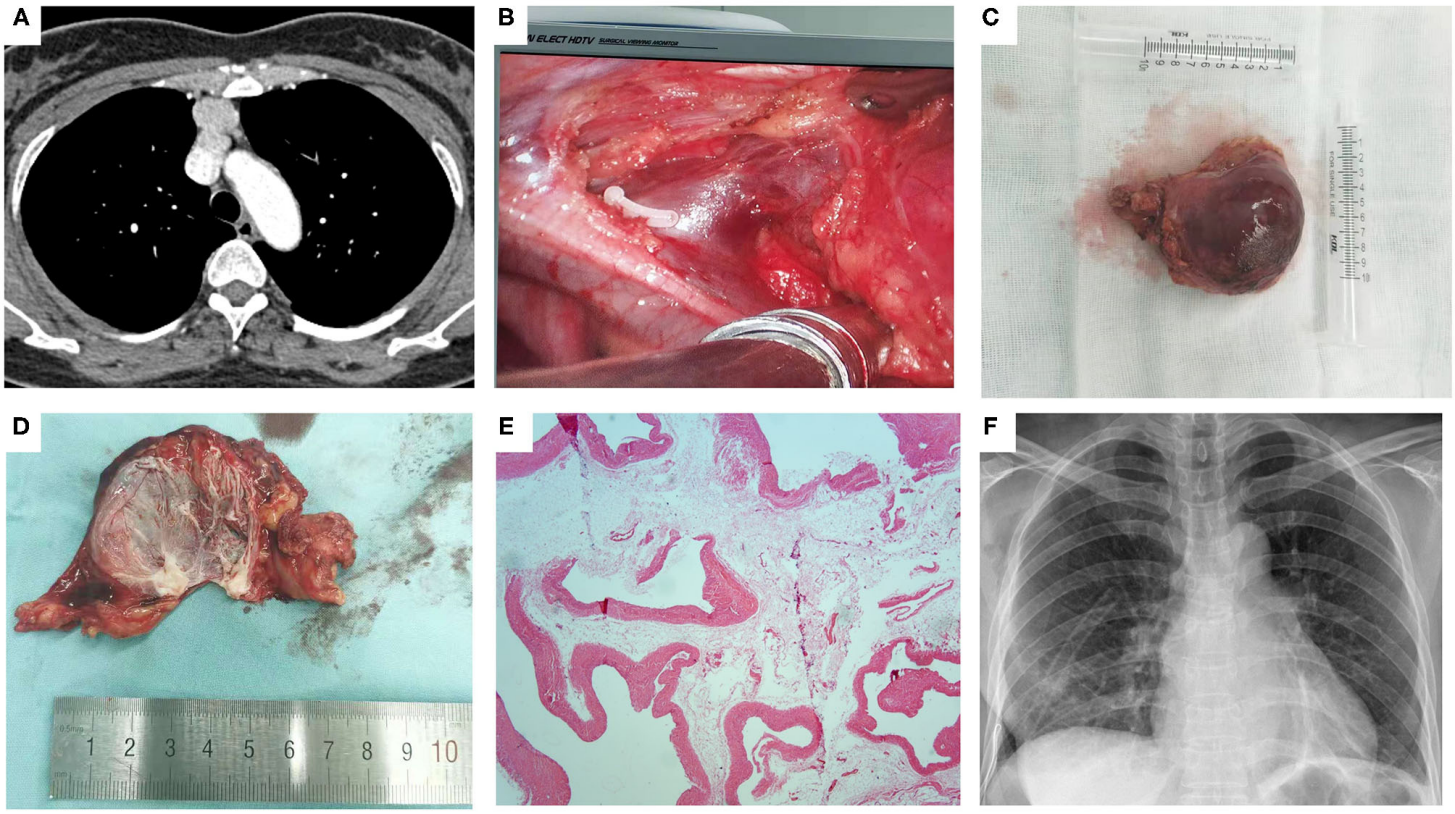
The left innominate vein aneurysm. **(A)** Contrast-enhanced CT of the chest showed a 4.8 × 4.6 cm anterior mediastinal mass with significant homogenous enhancement after injection of the contrast; **(B)** Intraoperative view of the root of the aneurysm after dividing the left internal thoracic vein with a vascular clamp; **(C)** Gross appearance of the aneurysm; **(D)** Gross appearance of the cut-up aneurysm after the blood being flowed away; **(E)** Postoperative pathology confirmed the diagnosis of the left innominate vein aneurysm; **(F)** Chest radiography on postoperative day 1 showed complete reexpansion of the lung, with no obvious pneumothorax or hydrothorax.

A 50-year-old female patient presented with the incidental finding of an anterior mediastinal mass on chest radiography during a routine health examination in October 2020. No related subjective symptoms were reported and the physical examination showed no abnormalities. Contrast-enhanced computerized tomography (CT) of the chest showed a 4.8 × 4.6 cm anterior mediastinal mass with significant homogenous enhancement after injection of the contrast (mean CT values before and after contrast injection were 38 and 115 HU, respectively), suggesting a diagnosis of Castleman's disease, but not excluding thymoma (Figure 1).

The patient underwent surgical resection of the anterior mediastinal mass under a thoracoscopic approach. After the intubation of the single lumen tube with a bronchial obstruction balloon, single lung ventilation on the contralateral side was performed under general anesthesia. The patient was placed in the lateral position with a retroversion of 30°. The operation was performed via a single-utility port: the endoscopic port was a 1-cm incision at the 6th intercostal space in the midclavicular line, and the utility port was a 3-cm incision at the 4th intercostal space along the anterior axillary line. Intraoperatively, the anterior mediastinal mass was determined to be an aneurysm originating from the left innominate vein. Through careful surgical manipulations and adequate exposure of the operative field, the root of the aneurysm was fully dissected and ligated, and then the root vessel was divided with a cutter closure (Supplementary Video 1). Postoperative pathology confirmed the diagnosis of left innominate vein aneurysm. The patient experienced an uneventful postoperative course. The thoracic drainage tube was removed on postoperative day 1 after chest X-ray imaging showed complete re-expansion of the lung, with no obvious pneumothorax or hydrothorax. She was discharged from the hospital on postoperative day 2. At her 6-month follow-up, the patient did not report any specific discomfort.

## Discussion

Mediastinal venous aneurysms are extremely rare and only a few cases have been reported in the literature, most of which occur in the superior vena cava (1). Mediastinal vein aneurysms are classified into fusiform and saccular types (3). The etiology of mediastinal vein aneurysms are unknown and may be caused by congenital malformations, trauma, degeneration, inflammation, infection, and tumor (4). Isolated left innominate vein aneurysms may be asymptomatic and are often discovered incidentally during routine chest imaging. However, they are sometimes detected due to the occurrence of complications provoked by the aneurysms (1). If the aneurysm is large and compresses surrounding structures, non-specific symptoms such as palpitation and dyspnea may occur (2).

Left innominate aneurysms are most often identified on CT and may be difficult to distinguish from other anterior mediastinal masses such as thymoma (5). It is important to consider the possibility of this condition when a homogenously-enhanced anterior mass with uniform attenuation is detected, especially if it is not clearly bordered by a large blood vessel or if a venous connection appears present (6). Unnecessary needle biopsies, which may cause serious complications, need to be avoided at this time. It has also been suggested that the contrast medium should be injected through the left antecubital vein to facilitate a clearer visualization of the aneurysm (6). If a venous aneurysm is suspected or cannot be excluded by CT, magnetic resonance imaging, CT angiography or invasive venography may be performed to confirm the diagnosis (2). A correct diagnosis is requisite for making appropriate treatment decisions.

Treatment modalities for mediastinal venous aneurysms include endovascular intervention, observation, and surgical resection. The role of endovascular interventions for mediastinal venous aneurysms is unclear (7). Follow-up observation alone may be sufficient for the treatment of very small asymptomatic venous aneurysms. The choice of surgical treatment depends to a large extent on the location of the aneurysm and the balance between the potential consequences of the aneurysm itself and the risks of surgical intervention. Considering the low complication rate of venous thrombus embolism (VTE) in thoracic venous aneurysms and the high risk of surgical resection, Teter et al. (7) recommended observation with regular CT follow-up as the preferred treatment modality. Surgical intervention is required if the mass is enlarged and becomes compressed, or if there is a risk of pulmonary embolism, vessel rupture, or complete venous obstruction due to venous compression secondary to intravascular blood clot formation (8).

No specific guidelines exist guiding how to select the appropriate surgical approach when the resection of the aneurysm is required. It has been suggested that median sternotomy is the best surgical approach due to the ease of controlling bleeding, revascularization, and removal of the embolism during surgery (5). Fang et al. (2) described a case of left innominate vein aneurysm treated with venous reconstruction of the lateral wall of the superior vena cava and left innominate vein by autologous pericardium under median sternotomy. Gozdziuk et al. (9) report a case of superior vena cava aneurysm resected through anterolateral thoracotomy. It is known that the main advantage of robotic-assisted surgery is the remarkably free movement of joint-equipped robotic forceps under three-dimensional high-vision, which allows surgeons to perform precise manipulations around critical structures in a narrow space such as the anterior mediastinum (10, 11). The robotic-assisted thoracic approach may be feasible for some well-selected, uncomplicated aneurysms that simply require intraoperative dissection and tangential resection (12). Herein, we describe a rare case of isolated left innominate vein aneurysm presenting as an anterior mediastinal mass. The patient was preoperatively misdiagnosed with Castleman's disease, without excluding the possibility of thymoma and underwent successful surgical resection of the aneurysm under the thoracoscopic approach.

In summary, we believe that this report emphasizes the necessary awareness of thoracic surgeons that left innominate vein aneurysm should be included in the differential diagnosis of anterior mediastinal mass. Thoracoscopic resection of the left innominate vein aneurysm is feasible under the hands of an experienced surgeon. Careful intraoperative handling and adequate exposure of the operative field are prerequisites for a successful surgical intervention. Undoubtedly, minimal surgical trauma contributed to the patient's rapid post-operative recovery. Nevertheless, we need to aware that open thoracotomy or median sternotomy should be selected as the first choice for surgeons who lack sufficient experience in thoracoscopic surgery, with the aim of avoiding intraoperative accidents.

## Data Availability Statement

The original contributions presented in the study are included in the article/Supplementary Material, further inquiries can be directed to the corresponding author.

## Ethics Statement

The studies involving human participants were reviewed and approved by Clinical Research Ethics Committee of the Second Xiangya Hospital. The patients/participants provided their written informed consent to participate in this study. Written informed consent was obtained from the individual(s) for the publication of any potentially identifiable images or data included in this article.

## Author Contributions

YH drafted and edited this manuscript, assisted in the surgery, and analyzed the patient data. SR edited this manuscript and analyzed the patient data. CZ, JL, LW, PX, and FY analyzed the patient data. MZ assisted in the surgery. WL performed the surgery, edited this manuscript, and analyzed the patient data. All authors have read and approved the final manuscript.

## Funding

This work was supported by National Natural Science Foundation of China (81972638 and 81972195), Natural Science Foundation of Hunan Province, China (2019JJ30038), the Hunan Provincial Key Area R&D Program (2019SK2253), the Scientific Research Program of Hunan Provincial Health Commission (20201047), and the Clinical Medical Technology Innovation Guide Project of Hunan Province (S2020SFYLJS0311).

## Conflict of Interest

The authors declare that the research was conducted in the absence of any commercial or financial relationships that could be construed as a potential conflict of interest.

## Publisher's Note

All claims expressed in this article are solely those of the authors and do not necessarily represent those of their affiliated organizations, or those of the publisher, the editors and the reviewers. Any product that may be evaluated in this article, or claim that may be made by its manufacturer, is not guaranteed or endorsed by the publisher.
